# Implementation of IMS/NGN Transport Stratum Based on the SDN Concept

**DOI:** 10.3390/s23125481

**Published:** 2023-06-10

**Authors:** Sylwester Kaczmarek, Maciej Sac, Kamil Bachorski

**Affiliations:** 1 Faculty of Electronics, Telecommunications and Informatics, Gdańsk University of Technology, Narutowicza 11/12, 80-233 Gdańsk, Poland; 2 ADVA Optical Networking, Łużycka 8C, 81-537 Gdynia, Poland

**Keywords:** IMS, NGN, ONOS, SDN

## Abstract

The paper presents the development and verification of software and a testbed aiming to demonstrate the ability of two telecommunication network concepts—Next Generation Network (NGN) and Software-Defined Networking (SDN)—to cooperate. The proposed architecture includes components of the IP Multimedia Subsystem (IMS) in its service stratum and of the SDN (controller and programmable switches) in its transport stratum, providing flexible transport resource control and management via open interfaces. One important feature of the presented solution is the inclusion of ITU-T standards for NGN networks, which are not considered in other related works. The paper includes details regarding the hardware and software architecture of the proposed solution as well as results of the performed functional tests, which confirm its proper operation.

## 1. Introduction

Due to the significant amount of new and dynamically changing traffic (concerning distance learning, remote work, remote handling of official matters, etc.), the COVID-19 pandemic significantly increased the demand for flexible transport resource control and management mechanisms in telecommunication networks. These requirements can be fulfilled using the Software-Defined Networking (SDN) concept [1,2,3,4,5], which makes it possible to increase the level of network automation via programmable resource control and management. This is facilitated by separating the control plane from the data plane within network devices. According to this concept, SDN controllers that manage network resources operate within the control plane. The data plane contains programmable switches, which can be devices based on various technologies, such as SD-WAN [6]. The SDN architecture ensures the controller and switch solutions, as well as the protocols used for communication between these devices, are open. The Open Networking Operating System (ONOS) [7] and Floodlight [8] are two of the most popular SDN controllers, while in the SDN concept, the OpenFlow protocol [9] is used in most cases for exchanging information between the control and data plane in order to manage and control flows.

The SDN controller is a central component responsible for resource control in the supported network of programmable switches. Centralized transport resource control functions also form the basis of the Next Generation Network (NGN) [10] concept. The basic reference model of the NGN architecture is developed by the International Telecommunication Union Telecommunication Standardization Sector (ITU-T). The NGN network consists of two strata. The service stratum is based on the IP Multimedia Subsystem (IMS) architecture components [11], which are also an important part of the 4G and 5G mobile networks and use the SIP [12] and Diameter [13] communication protocols, among others. The architecture is referred to as the IMS/NGN network, as the IMS is applied in the NGN service stratum. The main function of the IMS is the Call Session Control Function (CSCF) servers. They control end user sessions, which can be terminated in the IMS/NGN network or other networks. They also provide service-related information to the transport stratum, namely to the Resource and Admission Control Function (RACF) unit, via the Diameter communication protocol. RACF is the central network resource control point, capable of interpreting received Diameter protocol messages as well as managing and controlling transport stratum resources.

After analyzing the SDN and IMS/NGN architectures and functionalities, an integration concept and its rules were proposed. This proposal assumes the SDN controller in the transport stratum of the IMS/NGN network will be used as the component controlling the programmable switches as network resources. This requires preserving the RACF component’s functionality of interpreting Diameter messages received from the Proxy-CSCF (P-CSCF) and translating them into the API of the selected SDN controller.

The aim of this paper is to describe the implementation and testing process for the above-mentioned solution. The developed software and testbed prove that applying the SDN concept in the IMS/NGN network transport stratum is possible and may increase the automation of transport resource control and management. It is worth mentioning that the described solution takes into account ITU-T NGN standards, which are not considered in other related works. The preliminary results of our work on integrating the SDN and IMS/NGN concepts were described in the conference paper [1]. The extended research on this topic is presented in detail in this paper, which is organized as follows. Section 2 contains a review of related work. Section 3 describes the proposed architecture of the IMS/NGN network using the SDN concept in the transport stratum and details on the cooperation of these concepts. It also contains the steps necessary to implement the proposed integration of SDN and IMS/NGN. Section 4 provides details on the practical implementation of the solution based on open-source software and the resulting difficulties and consequences. It also contains the results of the performed functional tests. The paper is summarized in Section 5 where further work planned is also indicated.

## 2. Related Work

The purpose of this review was to identify current research works concerning the practical implementations of cooperation between the SDN and IMS/NGN concepts, especially those that take into account both service and resource control procedures. During the review, the following features were especially relevant: taking into account ITU-T standards for NGN networks, available results of functional and performance tests, access to the source code of the software (which can be further used and modified).

The review showed that work is underway to integrate and ensure the cooperation of the IMS/NGN and SDN concepts; however, no papers that fully met the above-mentioned requirements were found. In some of the works, only the network architecture and service scenarios concepts were proposed, but these concepts were not implemented and tested in practice, e.g., Ref. [14]. Other works focused on resource control (OpenFlow protocol) and did not consider service control (SIP and Diameter protocols), e.g., Ref. [15].

Moreover, several scientific papers were found concerning the implementation of the IMS network cooperating with the SDN concept. The common features of these solutions are a focus on the pure IMS architecture (without considering the ITU-T NGN standards) and a lack of access to the source code. An example is the OSIMS project carried out at the University of Patras [16,17]. The authors prepared a test environment in which they integrated the functionality of the IMS and SDN networks and implemented communication between them. They used the Open IMS Core project to implement the core functionality of the IMS network. The SDN network was implemented using the Floodlight controller, which controls programmable Open vSwitch switches. Several projects similar to OSIMS were found, even using the same sets of open-source software, e.g., Refs. [18,19].

Ref. [20] provides a different approach to the cooperation of IMS and SDN concepts. In the proposed architecture and service scenarios, the SIP Application Server (SIP AS) mediates every multimedia session and uses the REST API to communicate with the SDN controller and manage the flow tables in the programmable switches. Such an approach does not conform with ITU-T standards [10], which specify that the P-CSCF server is responsible for communication with the resource control unit (RACF).

The most important features of the above-mentioned related works are summarized in Table 1. The consecutive columns of this table have the following meaning:

- Work: reference number;
- Concept implemented: “yes” when the work concerns a practical implementation, “no” when only a concept is presented;
- Service control: “yes” when service control elements from IMS are included, “no” otherwise;
- ITU-T NGN standards: “yes” when ITU-T standards for NGN networks are included, “no” otherwise;
- Source code available: “yes” when the project source code is obtainable, “no” otherwise.

Summarizing the presented considerations, no solutions were found concerning the cooperation of IMS/NGN and SDN that meet all the assumed requirements expressed in columns 2–5 of Table 1. This fact led to the implementation of a project of integrating the IMS/NGN and SDN concepts at the Gdańsk University of Technology—GUT (last row of Table 1), which is described in this paper and overcomes the drawbacks of the other existing solutions. The GUT project is based on open-source software, which is partly different to those utilized in Refs. [16,17,18,19]. The Open Networking Operating System (ONOS) [7], developed by the Open Networking Foundation (ONF) consortium, was used as the SDN controller. The selected functionality of the IMS network was implemented using the CDiameterPeer [21] application, which generates Diameter protocol messages, among others. The CDiameterPeer application is a part of the Open IMS Core project, which enables further integration with the project’s software. In the solution implemented at GUT, as in the case of Refs. [16,17,18,19], Open vSwitch [22] functions as a cluster of programmable switches.

## 3. Application of SDN in the Transport Stratum of the IMS/NGN Network

This section contains a proposal of an integrated (IMS/NGN and SDN) network architecture and resource control scenarios. It is the basis for the work on the practical implementation of the integrated network, whose details are provided in Section 4.

### 3.1. Architecture of the IMS/NGN Network Using the SDN Concept

**Figure 1:**
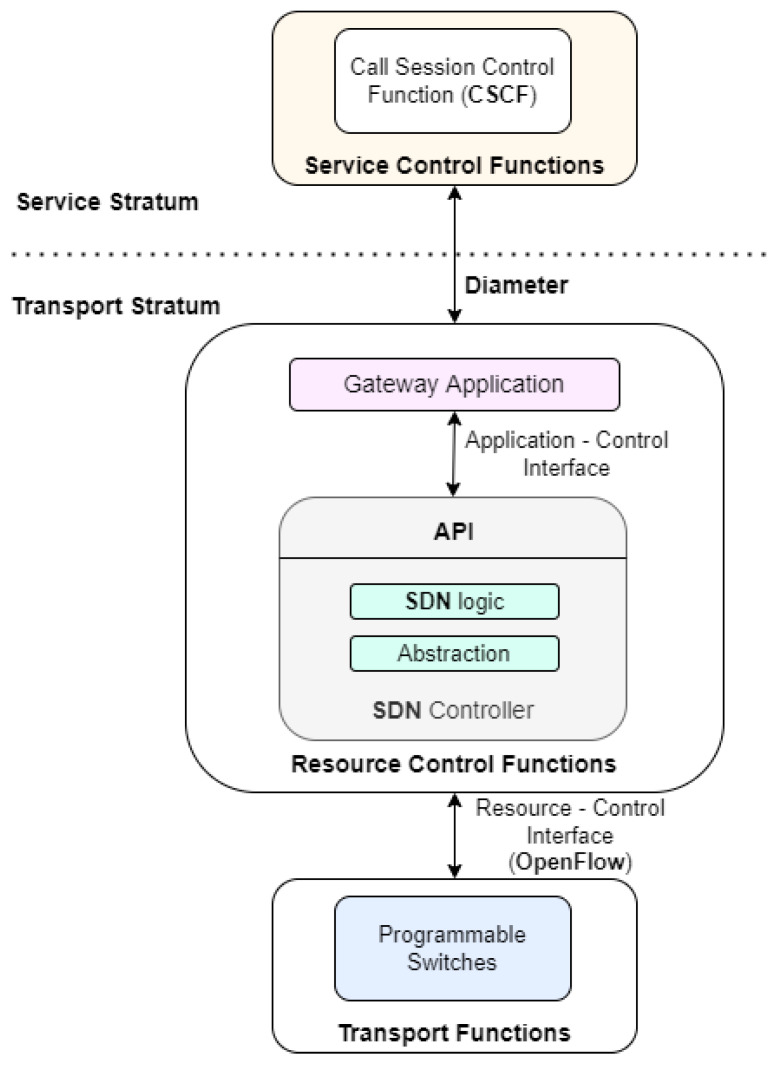
Proposed IMS/NGN architecture using the SDN concept in the transport stratum.

The first task of this project was to propose the integrated network architecture, including the IMS/NGN and SDN elements, which is depicted in Figure 1.

From this project’s point of view, the most important element of the IMS/NGN network service stratum is the set of CSCF servers, especially the P-CSCF server. It sends Diameter protocol messages determining the parameters of the requested services to the transport stratum. The recipient of these messages is the RACF unit, visible from the service stratum as a Gateway Application (GA). The GA functionality includes interpreting Diameter protocol messages and then calling the API of the SDN controller, which properly controls the network resources. The Gateway Application and the SDN controller (with SDN control logic and abstraction functionality) are included in the proposed architecture as the resource control functions and are located in the transport stratum. These functions use the OpenFlow protocol to communicate via the resource–control interface with the transport functions, which include programmable switches.

It should be mentioned that the interface between the P-CSCF and the Gateway Application is standardized [23]. Therefore, it can be implemented using available applications supporting Diameter protocol communication after appropriate source code adaptation. A similar situation exists for the resource–control interface, which exchanges information using the OpenFlow protocol [9]. The only interface which is not standardized is the application–control interface between the Gateway Application and the SDN controller.

### 3.2. Service Scenarios in the Integrated Network

**Figure 2:**
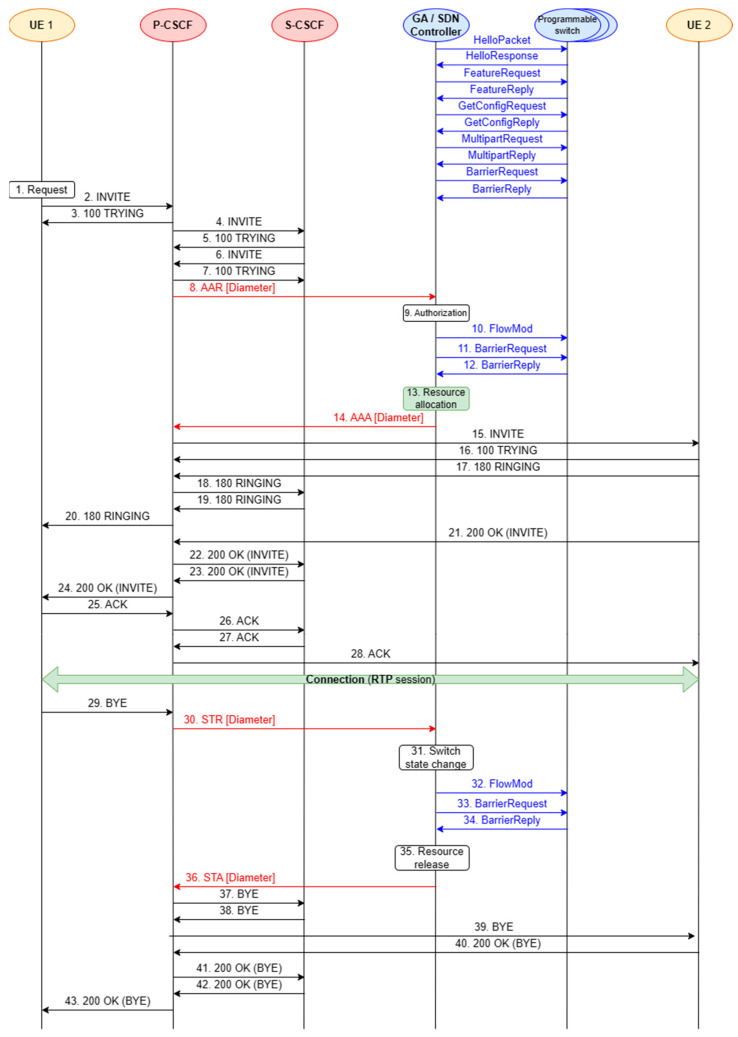
Service scenario for a successful call (transport resources available).

**Figure 3:**
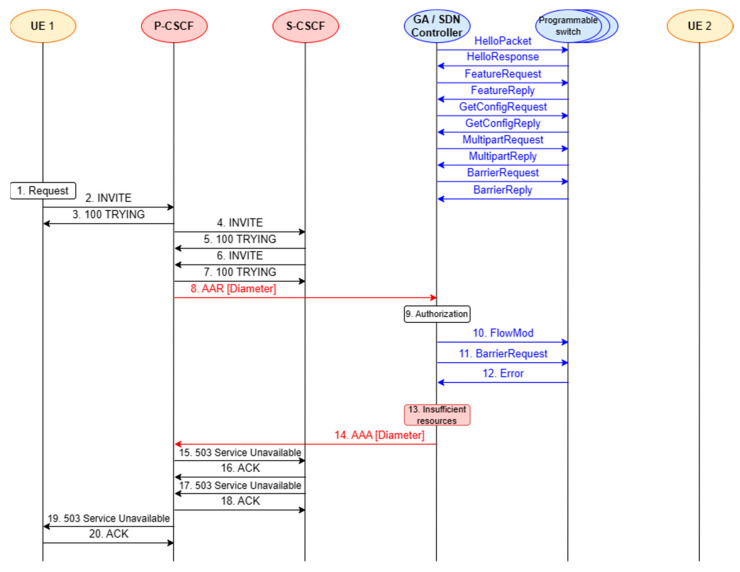
Service scenario for an unsuccessful call (transport resources unavailable).

The proposed network architecture considers two service scenarios. They take into account different states (availability and unavailability) of the transport resources and are presented in this subsection (Figure 2 and Figure 3). The functionality of the Gateway Application, which integrates the concepts of IMS/NGN and SDN, is implemented based on these scenarios.

The prepared service scenarios provide a better overview of the stages of software implementation and testing, which are described in the next section. The first scenario assumes a successful call set-up between two end devices in the IMS/NGN network (Figure 2). The case of insufficient transport resources, resulting in an unsuccessful call set-up, is considered in the second scenario (Figure 3).

In both scenarios, elements of the IMS network, such as P-CSCF and Serving-CSCF (S-CSCF), were included along with the end users (User Equipment—marked as UE 1 and UE 2 in Figure 2 and Figure 3). These elements communicate with each other using the SIP protocol. Moreover, elements of the SDN concept were included: the controller and programmable switches. Each scenario covers the end-to-end process of making a call, from generating a service request to reserving resources and releasing them when the call is disengaged. Not all the above-mentioned steps are performed when the transport resources for the call are insufficient. All the presented scenarios contain SIP (black arrows) and Diameter (red arrows) signaling messages. They also include the process of network resource control using the OpenFlow protocol (blue arrows) for operations on flow tables. The scenarios do not take into account invoking the SDN controller API by the Gateway Application because it is a non-standardized interface and dependent on the choice of controller software.

The initial OpenFlow protocol messages for both considered scenarios (unnumbered messages in Figure 2 and Figure 3) concern establishing communication between the SDN controller and programmable switches: negotiating the protocol version, checking the capabilities of the switches, obtaining their detailed configuration, etc. The most critical events occurring during the successful call set-up scenario (Figure 2) are as follows:

- UE 1 generates a call set-up request, which is passed to the P-CSCF, S-CSCF and P-CSCF again (messages 2–7);
- resource reservation for the call—exchange of Diameter messages between the P-CSCF and SDN controller (messages 8 and 14; this communication is performed via the Gateway Application not depicted in Figure 2 and Figure 3) and OpenFlow messages between the SDN controller and programmable switches (messages 10–12);
- UE 2 is notified about the call request. It rings and accepts the call (messages 15–21);
- SIP 200 OK (INVITE) message (acceptance of the call) is exchanged between the IMS servers (P-CSCF, S-CSCF—messages 22–23);
- UE 1 is notified that UE 2 accepts the call and sends a confirmation to UE 2 (messages 24–28);
- UE 1 and UE 2 are participating in a voice connection (RTP session);
- UE 1 generates a call disengagement request, which is passed to P-CSCF (message 29);
- allocated transport resources are released—Diameter and OpenFlow communication (messages 30–36);
- SIP BYE message (call disengagement request) is exchanged between the IMS servers (P-CSCF, S-CSCF—messages 37–38);
- UE 2 is notified about the call disengagement (message 39) and sends a confirmation to UE 1 (messages 40–43).

The unsuccessful call set-up scenario (Figure 3) begins similarly to the successful one (Figure 2). However, in this case, there are insufficient transport resources for the call, which is signaled by the SDN controller (message 14). The remaining SIP messages (messages 15–20) are used to inform UE 1 about this fact.

It should be emphasized that the implemented project focuses on the Diameter protocol communication between the P-CSCF and the SDN controller (via the API called by the GA), as well as the OpenFlow protocol communication between the SDN controller and the programmable switches, as they are crucial for checking the interoperability of the IMS/NGN and SDN concepts. For this reason, implementing the SIP protocol communication between user terminals and CSCF servers is not necessary and was not performed. As a result, the following network elements shown in Figure 2 and Figure 3 are implemented in the considered project: P-CSCF, SDN controller (supported by the GA), emulated network of programmable switches. The included communication procedures concern resource reservation (successful—messages 8–14 from Figure 2; unsuccessful—messages 8–14 from Figure 3) and resource release (messages 30–36 from Figure 2).

### 3.3. Mapping between Diameter and HTTP REST/JSON Message Fields

This subsection presents the Diameter and HTTP REST/JSON message fields, which are mapped by the Gateway Application (Table 2 and Table 3) during transport resource reservation and release procedures. In the presented mappings, it is assumed that the HTTP REST API of the ONOS controller [7] is used (the choice of this controller is justified later). It should be mentioned that the described mappings are not given in the standards and are not present in any of the related papers analyzed (Section 2). Therefore, they represent an original contribution of this paper, which is very important in implementing the GA.

The Gateway Application maps the Diameter messages exchanged over the Rs interface (between the P-CSCF server and GA) to the HTTP REST/JSON API messages (exchanged between the GA and ONOS controller) and vice versa. During resource reservation (Table 2), the Diameter message fields (called Attribute–Value Pairs, AVPs [13,23]) are mapped to the equivalent JSON fields carried in HTTP messages (HTTP POST and responses) [7]. For resource release (Table 3), the set of required ONOS API parameters is small and is passed in the request URI of the HTTP DELETE message. Thus, the Diameter AVPs are mapped to these HTTP URI parameters.

An asterisk (*) before the name of an AVP, JSON field or URI parameter (Table 2 and Table 3) indicates that this element is mandatory and must be present in a particular message. The following conclusions can be drawn from an analysis of Table 2 and Table 3:

1. There are several Diameter AVPs, which are not directly mapped to HTTP JSON fields or URI parameters:
  a. Diameter session identifier generated in P-CSCF (Session-Id);
  b. AVPs resulting from Diameter peers’ (P-CSCF, GA) IP addresses or domain parameters (Origin-Host, Origin-Realm, Destination-Realm);
  c. AVPs with constant values (Auth-Application-Id, Auth-Request-Type);
  d. other AVPs without ONOS API equivalents (Termination-Cause);
2. There are several Diameter AVPs, which are mapped to HTTP JSON fields or URI parameters (Authorization-Lifetime, Media-Component-Description). The Media-Component-Description AVP containing the requested QoS parameters and IP flows classifiers is very important for resource reservation. This AVP includes several other AVPs and has a very complicated structure [23]. Therefore, instead of presenting its example contents, a reference is given in Table 2;
3. The Result-Code AVP is set based on the HTTP response code;
4. The "deviceId" and "treatment": {"instructions":[…]} JSON fields (Table 2) must be present in HTTP POST messages sent from the GA to the ONOS controller for resource reservation. These parameters are related to the path in the programmable switches network whose resources should be modified—identifiers of switches and their output ports. As the service stratum does not provide these transport network topology parameters, they should be gathered by the GA;
5. For each successful resource reservation, the GA should store the Diameter Session-Id AVP, the identifiers of the related programmable switches and the identifiers of the flows created in these switches. During the resource release process, the service stratum provides the Session-Id AVP (in the STR message). Other identifiers must be determined by the GA and used in the "flowId", "deviceId" URI parameters for the HTTP DELETE message.

Section 3.4 describes the set of open-source software used to implement the integrated network functionality. As the chosen open-source software required extensive adaptation, the project implementation process was divided into stages.

### 3.4. Choice of Software and Stages of Implementation

The project began with selecting and integrating open-source software, implementing the required IMS/NGN and SDN network functionalities. The first piece of selected open-source software was CDiameterPeer [21], a peer-to-peer application providing the ability to send and interpret Diameter messages. Thus, it can be used to implement the functionality of P-CSCF (generating Diameter messages from the service stratum) and the Gateway Application component. The SDN network was built using several other open-source software solutions. The ONOS SDN controller [7] was chosen due to its extensive documentation and our experience from previous projects. This choice made it efficient to write the software code and to carry out functional tests of the cooperation of the IMS/NGN and SDN concepts, which was the aim of the paper. The controller manages network resources implemented by programmable switches (Open vSwitch [22]) emulated in the Mininet environment [24]. The communication over the SDN resource–control interface is based on the OpenFlow protocol.

The large amount of work related to implementing the project and its complexity required dividing the work into the following four stages (which will be described in more detail later in the paper):

1. Preparing the IMS/NGN and SDN environment (creating virtual machines, installing operating systems, configuring IP connectivity, installing the chosen open-source software).
2. Configuring the basic Diameter and OpenFlow communication (configuring the CDiameterPeer applications to provide the basic Diameter protocol communication between P-CSCF and GA—Figure 1; detailed standardized Diameter communication procedures will be implemented in the next stage; creating an emulated transport network in the Mininet environment and enabling its management by the ONOS controller using the OpenFlow protocol—Figure 1).
3. Implementing the interface with the Diameter protocol (implementing communication between P-CSCF and GA according to the ITU-T standards).
4. Implementing the interface with the HTTP REST/JSON protocol (implementing the GA functionality of translating Diameter protocol messages to the HTTP REST/JSON API messages of the ONOS controller for controlling transport resources).

## 4. Concept Implementation and Software Tests

**Figure 4:**
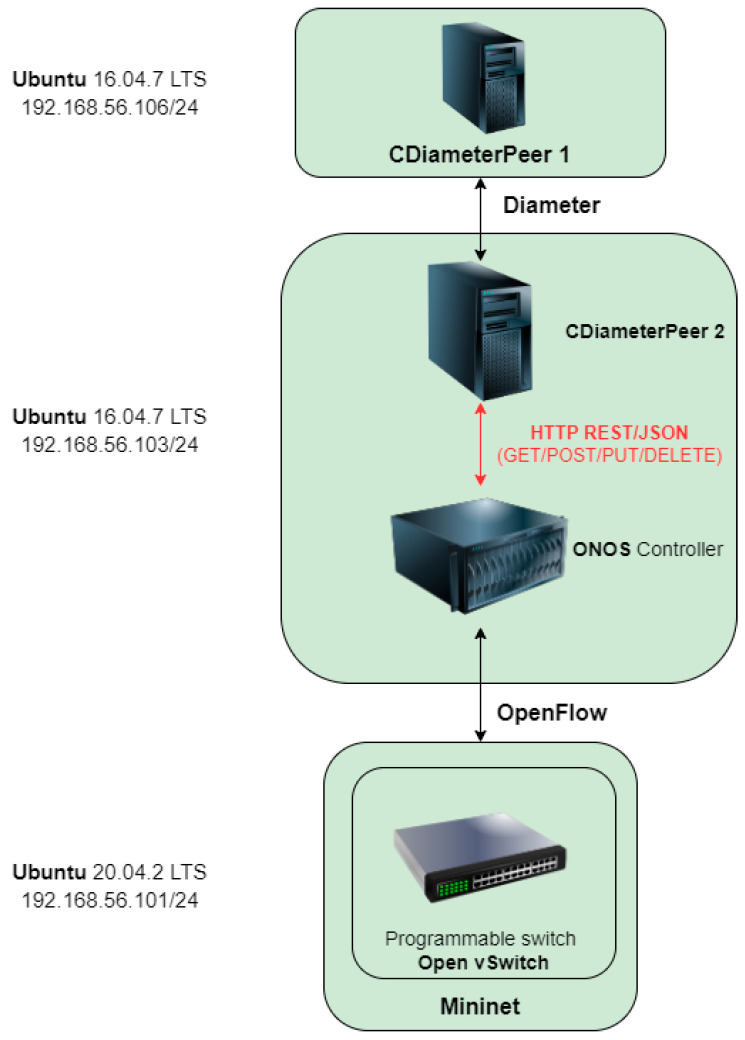
Architecture of the prepared environment.

Based on the proposed concept (Figure 1) and scenarios (Figure 2 and Figure 3), the implementation of the project began according to the architecture depicted in Figure 4. The consecutive stages of the performed work are presented in this section.

### 4.1. Preparation of the IMS/NGN and SDN Environment

The structure of the prepared IMS/NGN and SDN environment is illustrated in Figure 4. The experiments described below were run on a computer with an Intel Core i7-4790K processor (four cores, eight threads), 32 GB RAM, SSD drive and Windows 10 operating system. Three virtual machines running Ubuntu Linux distributions were created using the Oracle VM VirtualBox hypervisor. All machines were allowed to access the internet and an internal IP network, which was set up for their direct communication. Ubuntu distribution 16.04.7 LTS was installed on the first machine (CDiameterPeer 1). This system contains the CDiameterPeer software, which is used to generate Diameter messages and implements the selected functionality of the P-CSCF server. The second virtual machine contains the same version of the operating system and two open-source software solutions: CDiameterPeer, acting as a component of the Gateway Application (marked as CDiameterPeer 2), and the ONOS controller. The third virtual machine is based on the Ubuntu 20.04.2 LTS operating system and contains the Mininet software, which emulates the programmable switches implemented by Open vSwitch.

The created environment uses communication through three interfaces. The first is based on the Diameter protocol and is used for communication between CDiameterPeer 1 and CDiameterPeer 2. The HTTP REST/JSON protocol is used in the second interface for CDiameterPeer 2 to call the ONOS controller API. The OpenFlow protocol is applied in the third interface (interface of the SDN network) for the ONOS controller to manage the Open vSwitch programmable switches. The next subsection describes the process of configuring basic communication in the Diameter and OpenFlow interfaces.

### 4.2. Configuration of Basic Diameter and OpenFlow Communication 

Preparing the interface with the Diameter protocol required installing and running the CDiameterPeer 1 and 2 applications, as demonstrated in Figure 4. Both applications operate in a peer-to-peer topology by definition. They are written in C++ and compiled using GCC. A log file is created on startup by each application, which can be used to examine the operation of its individual processes.

Further configuration of individual Diameter peers involved modifying the configuration files stored in the XML format. For each peer, it was necessary to set the Fully Qualified Domain Name (FQDN) in the format of the IP address and the vendor ID of 11502 associated with the application ID of 16777235. According to the ITU-T standard [23], the last two values are used to indicate the Diameter Rs interface between the P-CSCF and RACF. For each Diameter peer, the XML configuration files also indicate the parameters of its neighboring peer: IP address and ISO/OSI layer 4 port.

**Figure 5:**
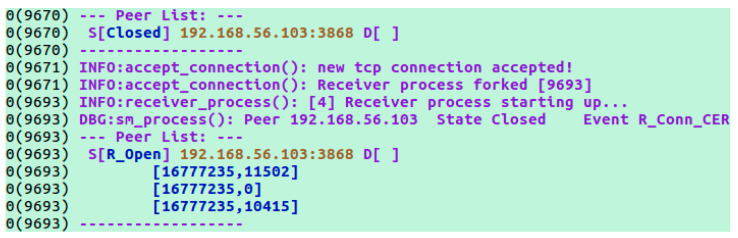
Log of CDiameterPeer 1 illustrating the establishment of a connection with CDiameterPeer 2.

**Figure 6:**
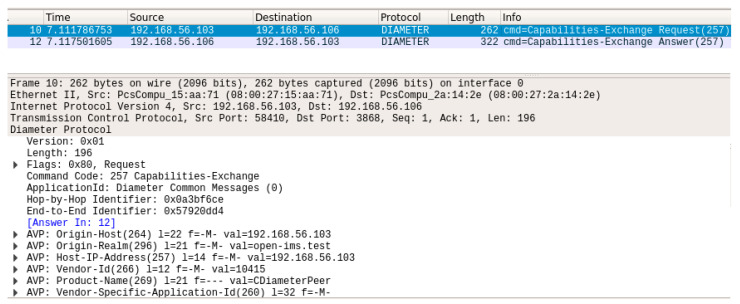
Diameter messages exchange when establishing a connection between peers.

After modifying the XML configuration files for both CDiameterPeer instances (CDiameterPeer 1 and 2), basic Diameter communication between the peers was established. The task was verified based on the CDiameterPeer application log for both instances (Figure 5), containing information about the established connection and open port 3868 (default Diameter port). The performed configuration was also confirmed using the Wireshark network sniffer. The exchange of the Capabilities-Exchange Request and Capabilities-Exchange Answer messages was captured (Figure 6), which are used to negotiate Diameter communication parameters (including the supported Diameter protocol applications).

In the next step, the OpenFlow interface was prepared (Figure 4). Initially, ONOS controller 2.6.0.b was installed from the GitHub repository. The installation required the following components of the Ubuntu operating system: Python modules, the Git version control system, tools to support the ZIP compression format, and OSGI libraries. It is worth mentioning that after installing all the necessary modules, problems were encountered during the compilation of the ONOS controller image, which were related to its cooperation with the installed version of the Ubuntu Linux system. The compilation failed on the latest versions of Ubuntu, i.e., 20 and 18. Eventually, the image was successfully built on Ubuntu 16. The described compatibility issues were not included in the ONOS controller documentation.

After building the ONOS controller image, it had to be configured. This involved using the provided CLI interface to set the IP address and launch the proper ONOS applications, among other things. These tasks are necessary for further work and enable, for example, the OpenFlow protocol support and activating the REST API. It was especially necessary to run the reactive relay application, i.e., the mechanism for installing flows on demand in the flow tables of programmable switches.

The second prepared element of the SDN network were the programmable switches (Open vSwitch 2.3.0), emulated in the Mininet 2.13.3 environment. The GitHub repository was used to install Mininet. Open vSwitch is built into Mininet by default. All that was required was to run it and check the status of the relevant processes from the Linux command line.

**Figure 7:**
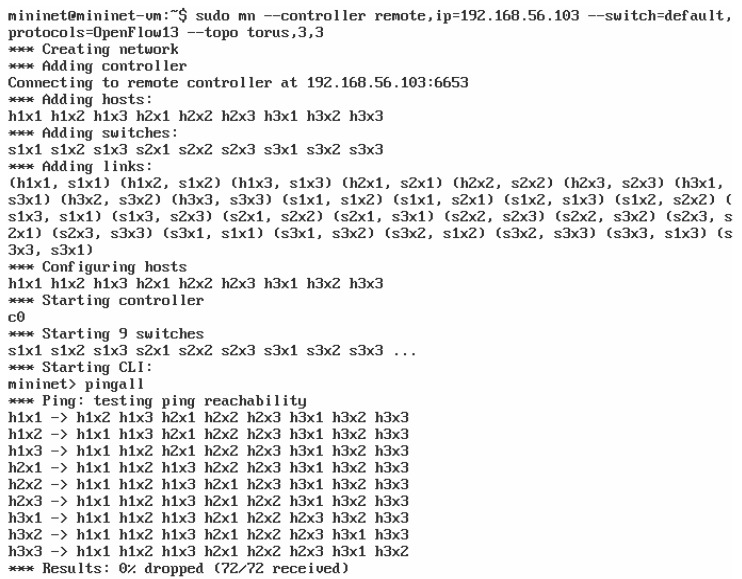
Mininet test result.

**Figure 8:**
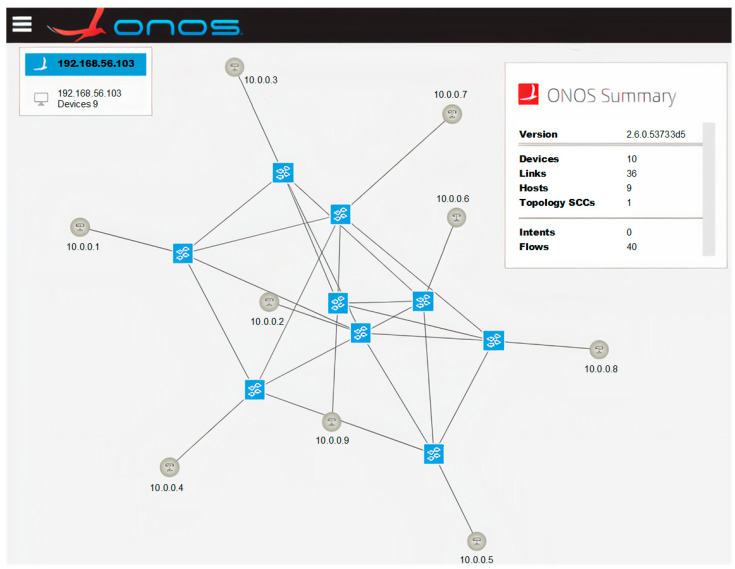
Topology of the network managed by the ONOS controller.

Open vSwitch and Mininet can be integrated with the ONOS controller by invoking a command in the Linux terminal (Figure 7, first two lines). For this operation to be successful, it is crucial to provide the appropriate parameters of the controller, the network of programmable switches and the communication between them. In the presented case (Figure 7), the mn command was run with arguments defining:

- IP address of the controller with the default port of 6653;
- OpenFlow protocol version 1.3 (using the latest supported version of 1.5, Mininet encountered problems cooperating with the ONOS controller, which were not reported in the documentation);
- target network topology (two-dimensional toroidal topology with a total number of nine switches; Figure 8).

**Figure 9:**
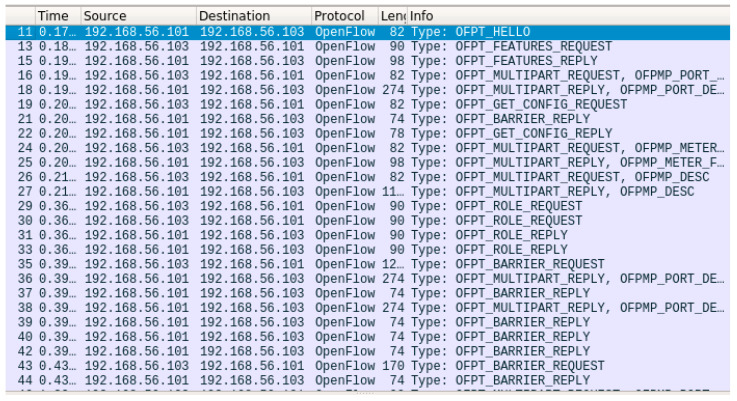
Packet dump on the OpenFlow interface.

After executing the mn command shown in Figure 7 (first two lines), the requested programmable switch network topology was created and successfully connected to the ONOS controller. Information about these events is provided in the Mininet log (Figure 7). In the next step, the Mininet CLI is started and used to verify the communication between the emulated switches (pingall command in Figure 7). The creation of the desired network topology was also monitored on the web interface of the ONOS controller (Figure 8). Finally, the OpenFlow protocol communication between the controller and programmable switches was captured using Wireshark (Figure 9). The communication process included, among others, confirming the protocol version used (Hello packets), determining the available switch ports (Features Request, Features Reply packets), obtaining detailed switch configurations (Get Config Request, Get Config Reply packets). The order of exchanging specific messages was as expected.

The results confirmed the correctness of the basic communication with the Diameter and OpenFlow protocols in their interfaces. The next subsection focuses on implementing the operation of the Diameter protocol interface in accordance with the ITU-T recommendation [23] for the Rs interface located between the P-CSCF and RACF.

### 4.3. Implementation of the Interface Running the Diameter Protocol

After implementing and testing the basic functionality of the Diameter interface, it was necessary to implement its full operation to control the transport resources in accordance with the ITU-T recommendation [23]. As illustrated in Figure 2, the following messages are exchanged over this interface:

- transport resources reservation requests—AA Request (AAR) messages;
- responses to transport resources reservation requests—AA Answer (AAA) messages;
- transport resources release requests—Session-Termination Request (STR) messages;
- responses to transport resources release requests—Session-Termination Answer (STA) messages.

The CDiameterPeer 1 virtual machine from Figure 4 generates individual types of Diameter requests (AAR and STR messages) in response to Unix signals sent to the main process of its CDiameterPeer application. For this purpose, the request_init() function was created in the main.c file of this application. Depending on the Unix signal type received (e.g., SIGUSR2), it generates an AAR or STR message with the Attribute–Value Pair (AVP) fields described by the ITU-T standard [23]. For example, for AAR messages, apart from the default AVP fields created by CDiameterPeer, other AVPs had to be added, including “Auth-Application-Id (258)”, “Auth-Request-Type (274)”, “Resource-Reservation-Mode (1003)” and “Destination-Realm (283)”. The second virtual machine in Figure 4 (containing the CDiameterPeer 2 software) required editing the server.c file of its CDiameterPeer application in order to process the AAR and STR messages. Its purpose is to call the REST API of the ONOS controller in response to the received Diameter messages (details are given in the next subsection) and to send the replies to these messages (containing the result of the transport resources reservation—AAA—or release—STA).

**Figure 10:**
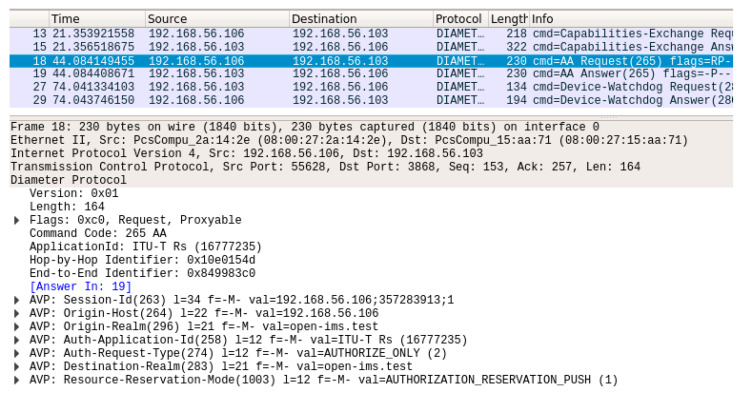
Diameter message dump for transport resource reservation.

**Figure 11:**
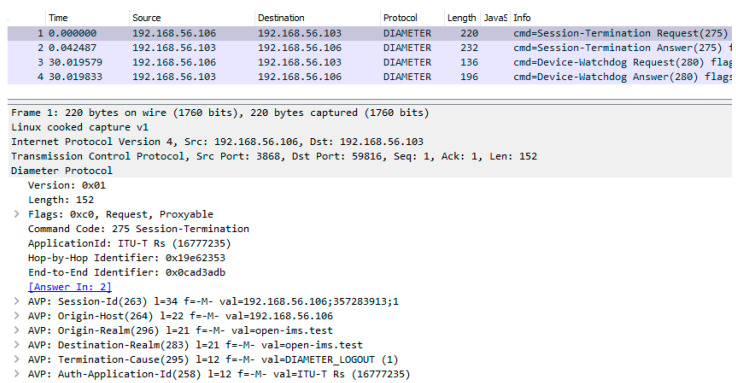
Diameter message dump for transport resource release.

The implementation of the Diameter interface was confirmed by analyzing the CDiameterPeer 1 and 2 logs and the Diameter message dumps created using the Wireshark packet sniffer for all kinds of generated requests. The captured Diameter messages related to resource reservation are presented in Figure 10. In addition to messages, which are characteristic of the Diameter protocol (Capabilities-Exchange Request/Answer, Device-Watchdog Request/Answer [13]), one can also see an AAR message containing standardized AVP fields and an AAA message, which is a response to the AAR. A Diameter message exchange concerning transport resource release is shown in Figure 11. It contains a resource release request (STR message with proper AVPs) and a response to this request (STA message). All the performed tests confirmed that the implementation of the Diameter interface in the described project is correct.

### 4.4. Implementation of the Interface with the HTTP REST/JSON Protocol

As already mentioned, in response to the received Diameter AAR and STR messages, CDiameterPeer 2 (the second virtual machine in Figure 4) generates HTTP REST/JSON messages to the ONOS controller to reserve or release transport resources. The operation of the interface with the HTTP REST/JSON protocol is implemented in the server.c file of the CDiameterPeer 2 application.

**Figure 12:**
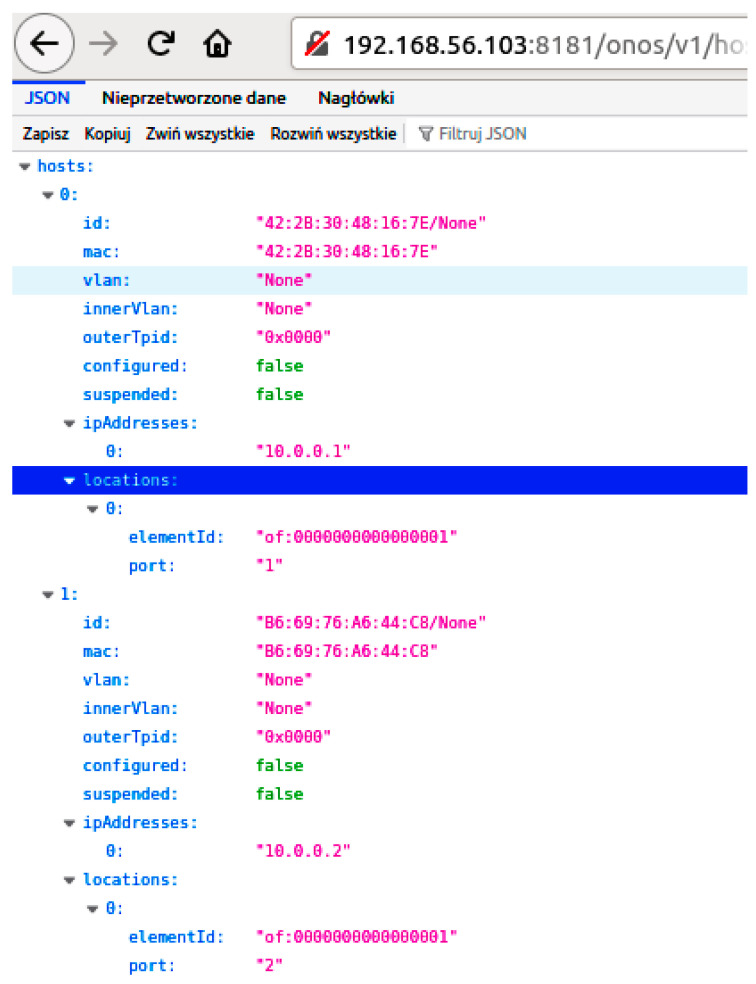
ONOS controller REST API testing.

**Figure 13:**
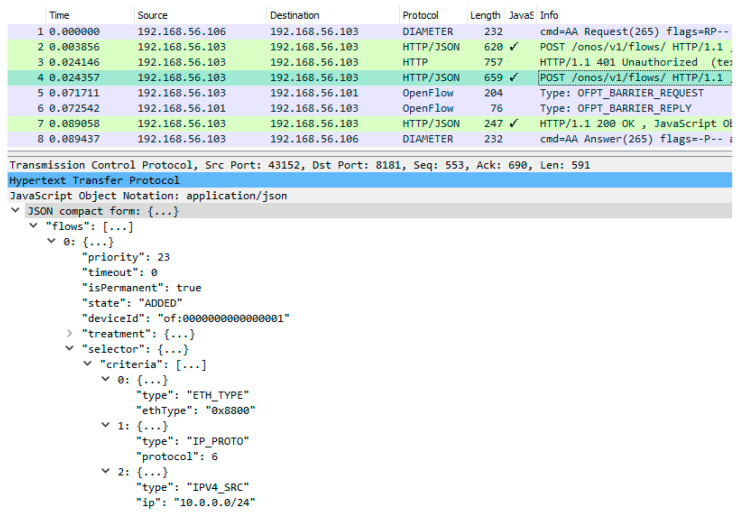
Complete message dump for transport resource reservation with selected content of the HTTP/REST API message (HTTP POST) sent to the ONOS controller.

**Figure 14:**
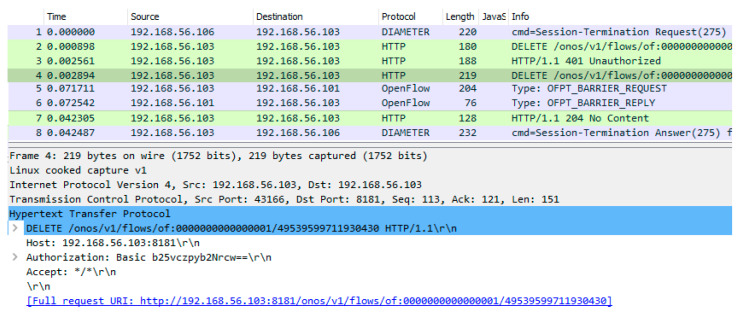
Complete message dump for transport resource release with selected content of the HTTP/REST API message (HTTP DELETE) sent to the ONOS controller.

In preparing this implementation, the REST API of the ONOS controller was investigated using a web browser, which can generate simple HTTP requests without a JSON body. A response to a request generated in this way is demonstrated in Figure 12. Based on this, the libcurl [25] (HTTP protocol) and JsonCpp [26] (JSON data exchange format) C++ libraries were used to implement the HTTP REST/JSON interface. The correctness of this implementation was confirmed based on a detailed analysis of message exchanges for complete resource reservation (Figure 13) and release (Figure 14) procedures. During the testing process, emphasis was placed on the contents of the messages sent over the HTTP REST/JSON interface.

**Figure 15:**
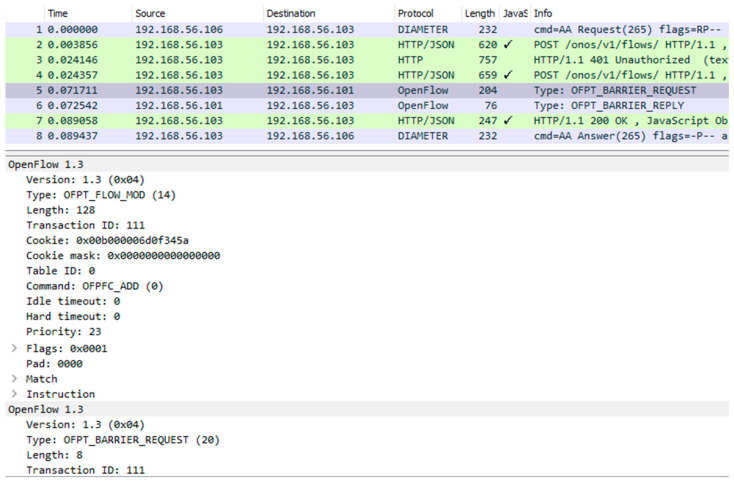
Selected content of the OpenFlow messages used by the ONOS controller to reserve transport resources (there are two messages in TCP packet no. 5—FlowMod and BarrierRequest).

It should be emphasized that Figure 13, Figure 14 and Figure 15 show the OpenFlow message exchange with only one programmable switch to be more compact. The communication with the other switches on the path is analogous.

**Figure 16:**
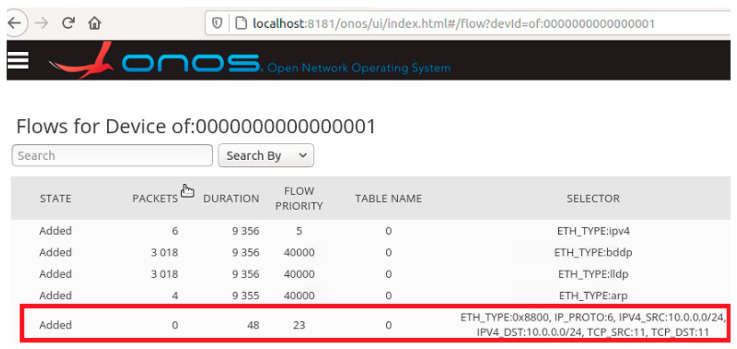
Flow table of a selected switch displayed using the ONOS controller GUI.

**Figure 17:**
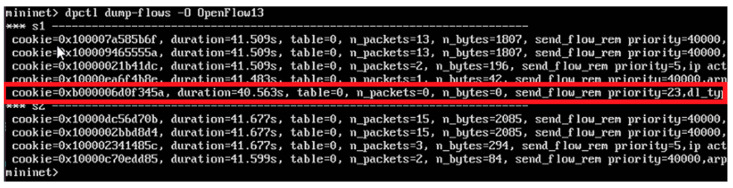
Flow table of a selected switch displayed using the Mininet CLI.

For transport resource reservation, an HTTP POST message (Figure 13) is sent from the CDiameterPeer 2 application to the ONOS controller. The parameters of the requested flow are described in the JSON body of this message, a fragment of which is presented in Figure 13. After receiving the HTTP POST message, the ONOS controller uses the OpenFlow protocol messages (the FlowMod and BarrierRequest messages are sent in one TCP packet—Figure 15) to modify the flow tables in the required switches. It then sends an HTTP 200 OK response to the CDiameterPeer 2 application, informing about the successful resource reservation (it contains the ID of the created flow). The success of the resource reservation can be confirmed by analyzing the flow tables in the switches involved, using the ONOS controller GUI and Mininet CLI, which now include the requested flow (it is marked using a red rectangle in Figure 16 and Figure 17). The same requested flow characteristics (among others, priority = 23 and cookie = 0x00b000006d0f345a) can be identified in Figure 13 and Figure 15–17.

For transport resource release, an HTTP DELETE message (Figure 14) is sent from the CDiameterPeer 2 application to the ONOS controller. The ID of the deleted flow is given in the request URI. The ONOS controller uses OpenFlow protocol messages to modify the flow tables in the required switches (the same set of messages is used as for resource reservation, but with slightly modified content) and sends an HTTP 204 No Content response to the CDiameterPeer 2 application, confirming the resource release. The HTTP response sent contains no information in its message body. The ONOS controller GUI and Mininet CLI were also used to confirm that the requested flow was deleted from the flow tables in the required switches, analogous to the way presented in Figure 16 and Figure 17.

### 4.5. Final Functional Tests

The previous subsections presented the stages of software development for the IMS/NGN network based on the SDN concept in the transport stratum. After each of these stages, the new functionalities and code fragments were tested. After verifying the correctness of the last stage described in Section 4.4, additional final tests were carried out. They concerned a detailed analysis of the signaling message flows for resource reservation and release, analysis of the content of these messages and the flow tables in the programmable switches.

As a summary of the final tests, comparisons of the obtained and assumed communication scenarios for transport resource reservation and release are presented in Table 4 and Table 5. A dash (–) in these tables means that HTTP REST/JSON API messages are not presented in Figure 2, as this figure is intended to be independent of the chosen controller software, while API messages are specific to particular controllers.

In addition to the comparisons presented in Table 4 and Table 5, during the final tests, a detailed analysis of the content of the transmitted messages and the verification of the flow tables in the programmable switches were also performed for both the resource reservation and release procedures. As presented in Figure 16 and Figure 17, the flow tables were checked using two methods: the ONOS controller GUI and the Mininet CLI. The final tests of the software developed under the project were fully successful and proved that it is possible to apply the SDN concept in the transport stratum of the IMS/NGN network, which will contribute to increasing the possibilities of programmable resource control and management.

## 5. Conclusions

The concept presented in this paper corresponds to the directions of work carried out in leading research centers and demonstrates the possibility of integrating modern telecommunication technologies, such as IMS/NGN and SDN. This is in line with the current effort put into optimizing management and control in telecommunication systems by increasing the level of automation. The possibility of using solutions from open-source projects is also very important here, as it reduces the implementation costs and shortens the time from concept to implementation.

The described project uses many open-source software solutions, implementing the specific functionality of IMS/NGN networks (CDiameterPeer) and SDN networks (ONOS controller with programmable Open vSwitch switches emulated in Mininet). In addition, tools were utilized that fit into the latest trends of using virtualization (Oracle VM VirtualBox hypervisor). This variety of open-source software and tools, as well as the deficiencies in the available documentation, require a high degree of proficiency in recognizing software configuration and modification problems.

Due to the large amount of work related to the implementation of the project and its complexity, the work was divided into four stages: preparing the IMS/NGN and SDN environment; configuring the basic Diameter and OpenFlow communication; implementing the interface with the Diameter protocol; and implementing the interface with the HTTP REST/JSON protocol. After completing each stage, the developed software was tested by analyzing the available logs and message dumps obtained using the Wireshark packet sniffer, among other things. Each successful test made it possible to start the next stage of project implementation. Additionally, when the whole implementation process was completed, a final detailed analysis of the message exchanges for resource reservation and release processes was performed. The results proved that the SDN concept can be successfully integrated and applied to cooperate with IMS/NGN networks.

The current project architecture (consisting of P-CSCF, GA, SDN controller and emulated network of programmable switches) was successfully used to achieve the aim of this paper, which was confirming the interoperability of the IMS/NGN and SDN concepts. Future work will include extending this architecture to investigate more complex services and service scenarios, which requires implementing the full IMS functionality in the service stratum. Functional and performance tests of such an extended architecture will be conducted. The results of performance tests are particularly important when considering industrial applications. The knowledge about the performance of particular system elements can be used, for example, to dynamically launch additional instances when the load increases in order to preserve low response times. Based on the results of the above-mentioned tests, we intend to propose analytical and simulation traffic models for the IMS/NGN network, similar to those proposed in Refs [27,28], but including the SDN concept in the transport stratum.

## Figures and Tables

**Table 1:** Comparison of the available approaches to cooperation between IMS/NGN and SDN.

Work	Concept Implemented	Service Control	ITU-T NGN Standards	Source Code Available
[14]	no	yes	no	not applicable
[15]	yes	no	no	no
[16,17]	yes	yes	no	no
[18]	yes	yes	no	no
[19]	yes	yes	no	no
[20]	yes	yes	no	no
this paper	yes	yes	yes	yes

**Table 2:** Mappings between Diameter and HTTP REST/JSON message fields for resource reservation.

Diameter Message	Diameter AVP (Code)	Sample AVP Value	HTTP Message	JSON Field	Sample JSON Field Value	Comment
AAR, AAA	*Session-Id (263)	192.168.56.106; 592269514;4				Diameter session Id generated in P-CSCF.
AAR, AAA	*Origin-Host (264)	192.168.56.106				IP address of Diameter message sender.
AAR, AAA	*Origin-Realm (296)	open-ims.test				Diameter origin realm name.
AAR	*Destination-Realm (283)	open-ims.test				Diameter destination realm name.
AAR, AAA	*Auth-Application-Id (258)	16777235				This AVP will always be set to 16777235 (standardized Id of the ITU-T Rs interface between P-CSCF and GA).
AAR, AAA	*Auth-Request-Type (274)	2				This AVP will always be set to AUTHORIZE_ONLY (2).
AAR, AAA	Authorization-Lifetime (291)	4294967295	POST	*"timeout"	0	Resource reservation holding time; the presented values mean “no timeout”.
AAA	*Result-Code (268)	2001	response to POST			Resource reservation result taken from the HTTP response code. For example, a successful reservation (HTTP 200 OK message) results in the AVP value of 2001.
			POST, response to POST	*"deviceId"	of:000000000000001	OpenFlow identifier of a programmable switch.
			response to POST	*"flowId"	281476241443288	OpenFlow identifier of a created flow.
			POST	"treatment":{"instructions":[…]}	{"type":"OUTPUT"," port": "1"}	Treatment instructions for the flow packets in a particular switch—forwarding to a specified output port.
AAR	Media-Component-Description (517)	refer to Ref [23]	POST	"selector":{"criteria":[… ]}	{"type":"ETH_TYPE", "ethType":"0x8800"}, {"type":"IP_PROTO", "protocol":6}, {"type":"IPV4_SRC", "ip":"10.0.0.0/24"}, {"type":"IPV4_DST", "ip":"10.0.0.0/24"}, {"type":"TCP_SRC", "tcpPort":1},{"type":"TCP_DST", "tcpPort":1}	Criteria for classifying packets to a given flow. They can be based on, among others, Ethernet frame type, IP transport protocol type, IP source and destination addresses and ports.

An asterisk (*) before the name of an element indicates that it is mandatory.

**Table 3:** Mappings between Diameter and HTTP URI parameters for resource release.

Diameter Message	Diameter AVP (Code)	Sample AVP Value	HTTP Message	URI Parameter	Sample URI Parameter Value	Comment
STR, STA	*Session-Id (263)	192.168.56.106; 592269514;4				Diameter session Id generated in P-CSCF during resource reservation (AAR message).
STR, STA	*Origin-Host (264)	192.168.56.106				IP address of Diameter message sender.
STR, STA	*Origin-Realm (296)	open-ims.test				Diameter origin realm name.
STR	*Destination-Realm (283)	open-ims.test				Diameter destination realm name.
STR	*Termination-Cause (295)	1				Reason for resource release. The most common value of 1 means resource release upon user request (call disengagement request sent to service stratum).
STR	*Auth-Application-Id (258)	16777235				This AVP will always be set to 16777235 (standardized Id of the ITU-T Rs interface between P-CSCF and GA).
STA	*Result-Code (268)	2001	response to DELETE			Resource release result taken from the HTTP response code. For example, a successful release (HTTP 204 No Content message) results in the AVP value of 2001.
			DELETE	*"flowId"	"281476241443288"	OpenFlow identifier of the flow, which needs to be removed from a particular flow table. It should be filled by the GA based on the Diameter session Id.
			DELETE	*"deviceId"	"of:000000000000001"	OpenFlow identifier of a programmable switch. It should be filled by the GA based on the Diameter session Id.

An asterisk (*) before the name of an element indicates that it is mandatory.

**Table 4:** Comparison of the obtained (Figure 13) and assumed (messages 8–14 from Figure 2) communication scenario for transport resource reservation.

Message No. in Figure 13	Message No. in Figure 2	Message Name	Comment
1	8	Diameter AAR (AA Request)	Resource reservation request sent from the P-CSCF to the GA.
2	–	HTTP POST	API request for adding a flow to the flow tables of the programmable switches sent from the GA to the ONOS controller (without authentication credentials).
3	–	HTTP 401 Unauthorized	API response indicating that authentication is required.
4	–	HTTP POST	API request for adding a flow to the flow tables of the programmable switches sent from the GA to the ONOS controller (with authentication credentials).
5	10, 11	OpenFlow FlowMod, BarrierRequest	OpenFlow messages sent from the ONOS controller to the programmable switches (FlowMod—request to add a flow; BarrierRequest—request to confirm the completion of the previous operations, which include adding a flow). These two OpenFlow messages are transported in one TCP packet (Figure 15).
6	12	OpenFlow BarrierReply	Confirmation of the completion of previous operations sent by the programmable switches to the ONOS controller.
7	–	HTTP 200 OK	API response indicating that a flow was successfully added, sent by the ONOS controller to the GA.
8	14	Diameter AAA (AA Answer)	Resource reservation response (success) sent by the GA to the P-CSCF.

**Table 5:** Comparison of the obtained (Figure 14) and assumed (messages 30–36 from Figure 2) communication scenario for transport resource release.

Message No. in Figure 14	Message No. in Figure 2	Message Name	Comment
1	30	Diameter STR (Session-Termination Request)	Resource release request sent from the P-CSCF to the GA.
2	–	HTTP DELETE	API request for removing a flow from the flow tables of the programmable switches sent from the GA to the ONOS controller (without authentication credentials).
3	–	HTTP 401 Unauthorized	API response indicating that authentication is required.
4	–	HTTP DELETE	API request for removing a flow from the flow tables of the programmable switches sent from the GA to the ONOS controller (with authentication credentials).
5	32, 33	OpenFlow FlowMod, BarrierRequest	OpenFlow messages sent from the ONOS controller to the programmable switches (FlowMod—request to remove a flow; BarrierRequest—request to confirm the completion of the previous operations, which include removing a flow). These two OpenFlow messages are transported in one TCP packet (analogous to Figure 15).
6	34	OpenFlow BarrierReply	Confirmation of the completion of previous operations sent by the programmable switches to the ONOS controller.
7	–	HTTP 204 No Content	API response indicating that a flow was deleted, sent by the ONOS controller to the GA.
8	36	Diameter STA (Session-Termination Answer)	Resource release response sent by the GA to the P-CSCF.

## Data Availability

Not applicable.
